# The Status of Fertility Preservation (FP) Insurance Mandates and Their Impact on Utilization and Access to Care

**DOI:** 10.3390/jcm13041072

**Published:** 2024-02-14

**Authors:** May-Tal Sauerbrun-Cutler, Allegra Rollo, Alexis Gadson, Jennifer L. Eaton

**Affiliations:** 1 Division of Reproductive Endocrinology and Infertility, Department of Obstetrics and Gynecology, Women and Infants Hospital and Warren Alpert Medical School of Brown University, Providence, RI 02903, USA; msauerbruncutle@wihri.org; 2 The Warren Alpert Medical School of Brown University, Providence, RI 02903, USA; allegra_rollo@brown.edu; 3 Shady Grove Fertility Center, Rockville, MD 20850, USA; gadsonak@gmail.com

**Keywords:** fertility preservation, oncofertility, cancer

## Abstract

Fertility preservation (FP) is the use of a specific medical intervention to protect the fertility of individuals whose disease or disease treatment may lead to infertility. These medical interventions include the cryopreservation of oocytes, embryos, ovarian tissue, sperm, and testicular tissue; oocyte and embryo cryopreservation are the most widely used interventions in the United States. Although guidelines recommend FP prior to undergoing gonadotoxic treatments, cost barriers are high. For example, the average cost of an oocyte cryopreservation cycle in the United States exceeds $10,000. High cost and lack of insurance coverage are two of the most cited reasons explaining the low Reproductive Endocrinology and Infertility (REI) referral rates and limited FP utilization. Broadening insurance mandates for FP prior to gonadotoxic treatments could improve utilization and provide cancer survivors with improved quality of life post treatment.

## 1. Background

The scientific literature regarding the impact of insurance mandates on fertility preservation (FP) across the United States remains limited due to the recent initiation of FP insurance mandates in 2017, evolving state FP regulations and insurance coverage criteria, and the limited number of patients accessing FP services. An electronic search was conducted on PubMed, with the key search words [fertility preservation, health care, insurance coverage, legislation, statute, mandate], to identify all relevant studies related to fertility preservation and state-issued insurance mandates published since 2017 (Appendix A) The objective of this literature review is to explore the primary literature and provide the present status of U.S. insurance mandates’ impact on FP utilization and access to care, with a focus on cancer survivors.

## 2. Introduction to Fertility Preservation

Fertility preservation is the use of a specific medical intervention, such as the cryopreservation of oocytes, embryos, ovarian tissue, sperm, and testicular tissue, to protect the fertility of individuals whose disease or disease treatment may lead to infertility [1]. The most widely used methods of fertility preservation in the United States are oocyte and embryo cryopreservation. Cancer survival rates have improved dramatically over the past few decades; however, many survivors are infertile secondary to the impact of their cancer treatments on their ovarian function. For example, survival rates for pediatric cancer patients have increased from 55% in the 1970s to 83% as of 2011 [2]. Therefore, it is even more important to offer patients opportunities to preserve their family-building options. The gonadotoxic effects of alkylating agents and pelvic radiation are well known. However, many newer cancer treatments have a less defined impact on fertility but also warrant consideration for FP consultations and procedures [3]. Fertility preservation consultations are performed by Reproductive Endocrinology and Infertility (REI) specialists. These FP consultations decrease long-term regret and improve quality of life scores in cancer survivors even if they decide not to pursue FP procedures. For these reasons, the American Society of Clinical Oncology (ASCO), American Society for Reproductive Medicine (ASRM), and European Society of Human Reproduction and Embryology (ESHRE) guidelines recommend that oncology and surgical providers counsel patients early regarding the possible gonadotoxic impact of cancer treatments on their fertility and refer them to REI specialists for FP procedures [4,5,6]. These guidelines are applied not only to cancer patients, but also to other patients whose treatments require the use of gonadotoxic agents, for example, the treatment of lupus and other autoimmune disorders. Despite the importance of FP and success of oocyte cryopreservation, a cross-sectional review of commercial insurance databases demonstrated that only 4.6% of reproductive-age oncological patients utilized FP procedures in 2016 [7]. Voigt et al. (2022) showed that only about 8% of women pursued fertility services after being diagnosed with cancer [8]. Meernik et al. suggested that only 1.2% of adolescent and young adult women diagnosed with cancer used FP [9]. Even though guidelines recommend FP prior to undergoing gonadotoxic treatments, cost barriers are high. The average cost of a cycle of in vitro fertilization in the United States, including procedures and medications but excluding storage, is $10,000 to $15,000 [10]. Oocyte and embryo cryopreservation are similarly, prohibitively expensive for many patients, while sperm cryopreservation is substantially less costly, averaging $745 for the first year, including storage [11]. The high cost and lack of insurance coverage for oocyte and embryo cryopreservation are two of the most cited reasons explaining the low REI referral rates and limited FP utilization [12,13].

## 3. Financial Hardship and Cost Barriers

The cost of an FP cycle is exceedingly high, with most patients unable to afford these critical procedures unless they have insurance coverage. The average procedure costs of one cycle of oocyte cryopreservation or embryo cryopreservation, excluding storage costs, are $10,000–$15,000 and $11,000–$15,000, respectively [14]. Many patients require more than one cycle, doubling or tripling these costs. Meernik et al. surveyed 65 women who had FP consultation and compared financial hardship between women with and without FP procedures. Financial hardship was defined as borrowing money, incurring debt, or filing for bankruptcy because of cancer, its treatment, or the lasting effects of that treatment [15]. The authors demonstrated that financial hardship was 1.5 times more likely among those who had FP procedures compared to those who did not. Furthermore, 12% of patients who underwent FP procedures reported debt of ≥$25,000 versus only 5% in the non-FP procedure group [15]. For this reason, women who have no insurance coverage are less likely to receive FP counseling by their providers [16,17,18,19]. Some providers believe it is unethical to discuss services that the patients will not be able to afford and attempt to protect patients from difficult decisions that may result in further financial hardship [18].

## 4. State FP Insurance Mandates

FP insurance mandates were created in 2017 to overcome cost barriers and increase access to care. Many states have already established infertility insurance mandates, but infertility mandates do not typically cover FP. Infertility insurance mandates cover infertility as a disease. FP insurance mandates cover FP in instances where a patient is not infertile, but rather, medical treatment for diseases such as cancer may prevent such patients from childbearing in the future. FP insurance mandates require insurance companies to cover FP services but vary in their requirements. Currently, there are 16 U.S. states with insurance mandates for FP, including California, Colorado, Connecticut, Delaware, Illinois, Kentucky, Louisiana, Maine, Maryland, Montana, New Hampshire, New Jersey, New York, Rhode Island, Texas, and Utah (Table 1). Massachusetts does not have an FP mandate but has a mandate to cover infertility, with most commercial insurers extending benefits to FP. There is heterogeneity in coverage between states with differences in benefits covered, definitions of what qualifies as medical FP, and exclusions based on patient age, company size, etc. [20]. For example, all 16 states specify that they will cover oocyte and sperm cryopreservation, but only 3 states mandate coverage for noncommercial plans such as Medicaid. Furthermore, no states mandate coverage for self-insured plans, and it is up to the employer to decide if they will offer this benefit. Self-insured plans are usually present in larger companies where the employers themselves collect premiums from enrollees and take on the responsibility of paying employees’ and dependents’ medical claims. Based on the 2021 Kaiser Family Foundation report, 64% of workers with employer-sponsored insurance have partially or fully self-funded plans [21], leaving many of their employees with no coverage for medical FP. Exemptions for self-insured plans derive from the Employee Retirement Income Security Act of 1974 (ERISA), which preempts the specific state regulation of self-funded insurance plans provided by private-sector employers [22]. Unfortunately, noncommercially insured patients, such as those with Medicaid, are also excluded from the insurance mandates in all but three states: Montana, Illinois, and Utah. Illinois provides a more expansive provision of services, noting that standard FP services are mandated in iatrogenic cases [23], while Montana and Utah provide a less expansive provision of services, specifying that the iatrogenic infertility must be caused by an active cancer diagnosis [24,25]. The reason that state FP insurance mandates do not include Medicaid patients is due to certain provisions in the Affordable Care Act. These provisions would require states to cover the additional costs [26] for FP, which is problematic for state budgets. Illinois has a provision in their standards for fertility coverage that exempt them from future decisions made by the U.S. Department of Health and Health Services that would require the state to pay for these benefits [23].

Connecticut and Rhode Island were the first two states to pass requirements for commercial insurers to cover FP in 2017. They differ in their mandated coverage, further illustrating heterogeneity in coverage between two states with mandates enacted around the same time. The Rhode Island mandate applies to all commercially insured patients regardless of employer size. In contrast, Connecticut does not cover individual commercial plans but only those with 50 or more employees. Advocacy is necessary to both expand FP insurance mandates and reduce exclusions to care in existing mandates. Legal work is currently active to try to reduce heterogeneity in coverage. Moreover, between 2017 and 2021, states took an average of 283 days to implement mandates, and most provided regulatory guidance after law implementation [19]. While it is important to provide insurance companies and regulators enough time to modify benefit plans, the lag between the enactment of laws and their effective implementation underscores the necessity of streamlining and clarifying the implementation process to provide benefits to patients as swiftly as possible.

## 5. Utilization Increases in States with FP Insurance Mandates

While FP insurance mandates are relatively new, FP consultation and procedure utilization are already starting to increase. Patel et al. evaluated data from the ASCO Quality Oncology Practice Initiative, which surveyed approximately 400 member clinics throughout the United States. States with insurance-mandated coverage for FP had significantly higher rates of fertility risk discussions compared with states without legislation (48.6% vs. 39.6%, *p* < 0.001) [27]. As part of the same study, patients’ data were evaluated from 1/2015 to 6/2019 at 448 practices across the country. In states with legislatively mandated coverage of FP, patients who were counseled about the risk of infertility were more often counseled regarding FP or were referred to a fertility specialist after the passage of mandated coverage (before legislation: 68 of 95 patients [71.6%]; after legislation: 21 of 26 patients [80.8%]). Although the time period post mandate was short in this study (two years after the first mandates in Rhode Island and Connecticut), the data are already confirming that once cost barriers are removed, utilization will steadily increase. More recent data from the only fertility center in RI demonstrated a more dramatic increase in utilization. The number of annual medical FP consults rose from 9 to 60 pre- and post-mandate (2021 data), respectively [28]. Forty five percent of patients who came for a medical FP consult between 2016 and 2021 proceeded to an FP procedure that included oocyte or embryo cryopreservation. These increases in FP consultations and procedures were primarily with commercially insured patients because these patients were covered by mandates. In contrast, only one noncommercial insurance patient pursued an FP cycle during the 2016–2021 time period [28]. In another study, the Society for Assisted Reproductive Technology (SART) database was utilized to investigate the increase in FP between 2016 and 2019 in the two states that implemented the first FP mandates. Comparing utilization in 2016 with 2019, medical FP cycle utilization increased by 108% and 86% in Rhode Island and Connecticut, respectively, compared to 64% in all other states. Even though this difference was not statistically significant (*p* = 0.5 (Rhode Island) and *p* = 0.2 (Connecticut)), the increasing trend shows promising results after only a year and a half since mandate implementation [29]. Similarly, all medically indicated FP consultations and completed cycles performed at one fertility center between 1 January 2017 and 31 December 2020 were collected, representing the two years before and after the enactment of the insurance mandate in Illinois. Illinois has a unique mandate in which both commercial and noncommercial insured patients have mandated medical FP coverage. Minimal changes in patient and cycle volume were seen in the first year after the insurance mandate took effect, but there were sharp increases in consultations (45%) and patients initiating FP procedures (46%) noted by year two compared to the year before the mandate was implemented. More patients with Medicaid insurance were seen after the enactment of the mandate (11 vs. 28), and a greater number of patients completed FP cycles (3 vs. 16, *p* = 0.003) [30]. This is the first study that identified the utilization of FP services in a state with a mandate that covers the Medicaid population. This type of research is critical to help advocate for mandates to expand coverage to all state constituents.

## 6. Utilization Increases with Infertility Insurance Mandates or Employer-Sponsored FP Coverage

Although the impact of FP mandates is difficult to quantify due to their recent passage, we can draw parallels from state-mandated insurance coverage for infertility. The utilization of FP or infertility treatment have been shown to increase when employers offer benefits or states mandate infertility insurance coverage. For example, when a hospital employer-based insurance started covering planned oocyte cryopreservation, the hospital-based fertility center saw a 79% increase in insured patients freezing oocytes, and the number of overall patients with hospital-based insurance coverage that underwent planned oocyte cryopreservation increased by a factor of 8 (5.3 to 41.5%, *p* < 0.001) [31]. Although the indications are different, we similarly expect a steep increase in medical FP utilization as coverage increases when more medical FP insurance mandates are passed. Survey studies also demonstrate that the primary concern regarding elective FP is cost. A survey of 171 female graduate students demonstrated that 59% of participants were mainly concerned about the cost of egg freezing; 81% indicated that they would be more likely to consider egg banking if it were covered by their insurance or paid for by their employer [32].

Infertility insurance mandates also increase access to care and can be used to predict how medical FP insurance mandates could change the proportion of patients who pursue procedures prior to cancer treatment. Jain et al. surveyed the proportion of IVF cycles performed in states with and without insurance mandates. Clinics in states that required complete coverage performed more in vitro fertilization cycles than clinics in states that required partial or no coverage (3.35 vs. 1.46 vs. 1.21 transfers per 1000 women of reproductive age, respectively; *p* < 0.001) [33,34].

## 7. Utilization Based on Demographic Characteristics

Certain racial/ethnic and demographic groups have historically underutilized FP services. Using the National Survey of Family Growth (NSFG), Voigt et al. demonstrated that household income, marital status, and race were significantly associated with women utilizing fertility services following a cancer diagnosis. Women who were married were more likely than those who were divorced/separated (OR 0.18, CI 0.07–0.49) or never married (OR 0.20, CI 1.03–1.10) to pursue fertility services. Hispanic (OR 0.32, CI 0.06–1.91) and non-Hispanic other (OR 0.18, CI 0.01–2.49) were less likely to utilize fertility services [8] than white patients. Meernik et al. linked a North Carolina cancer registry to SART to investigate the utilization of FP between 2004 and 2015. The study compared 96 women who utilized FP services, cryopreserving oocytes or embryos, to 7964 women who did not. In multivariable regression, women who were less likely to use FP were older at diagnosis (ages 25–29 vs. 35–39: OR = 6.27, 95%CI: 3.35, 11.73); non-Hispanic Black (vs. non-Hispanic white: PR = 0.44, 95% CI: 0.24, 0.79); and parous at diagnosis (vs. nulliparous: PR = 0.24, 95% CI: 0.13, 0.45); or lived in census tracts that were non-urban (vs. urban: PR = 0.12, 95% CI: 0.04, 0.37) or of lower socioeconomic status (quintiles 1–3 vs. quintiles 4 and 5: PR = 0.39, 95% CI: 0.25, 0.61) [9]. Future studies should identify if these disparities lessen once FP insurance mandates are in place. Insurance mandates that incorporate federal plans in their mandates will likely see an increase in utilization for patients with a lower socioeconomic status, while those that only include commercial insurance plans will see less of an increase. Beyond expanding insurance coverage, decreasing disparities in FP utilization necessitates ensuring that mandates have as few exclusions as possible. This will require patient and organizational advocacy through groups such as ASRM, RESOLVE, and The National Fertility Association to work with state legislatures and emphasize the medical necessity of these services.

As FP services become more common, states should consider that the use of these services by pediatric cancer patients will likely increase. Hong et al. described the expansion of an oncofertility program at a community pediatric hospital over three years, noting increases in oncofertility consultations from 6.7% (9/134 patients) in the first year to 40% (40/98 patients) by the third year [35]. In the third year, 11 patients elected further REI evaluations, sperm banking, and testicular tissue freezing [35]. Jackson Levin et al. highlighted the major financial concerns and economic distress experienced by adolescent and young adult cancer patients pursuing FP, with many patients relying on parental health insurance that may or may not cover FP services [36]. Beyond expanding FP coverage, the establishment and improvement of financial aid and counseling services are crucial in reducing financial toxicity and advancing more equitable access to FP services [36]. Patient navigators, including nurses, can be helpful in guiding patients through the complexities of insurance coverage, as well as assistance from philanthropic or financial organizations [37].

Of note, FP insurance mandates may have the potential to reduce disparities in geographic access to oncofertility care. Peipert et al. demonstrated that patients in states with FP legislation had higher geographic access to oncofertility centers compared to states without FP legislation, as defined by the percentage of patients within a 2 h travel time to a center [38]. Although research is needed to elucidate the contributing factors underlying this association, the authors posit that FP insurance mandates could create an economic incentive for an increase in oncofertility centers. As more states mandate FP, infertility, and employer-sponsored coverage, it is imperative to study the development of new oncofertility centers across the country, with an aim to preemptively address time and travel barriers for state centers with current or expected limited geographic reach.

The expansion of insurance mandates will ultimately necessitate obtaining a nuanced perspective of how cost, insurance coverage, and patient characteristics interact and affect FP utilization. Recent research underscores the value of integrating cancer registries with state-wide all-payer claim databases in synthesizing cancer patient and treatment characteristics with treatment costs [39,40]. By integrating claim databases, oncofertility studies can discover how cancer-related variables, including cancer type, treatment, and patient demographics, relate to FP-related variables, including procedures, consults, comorbidities, insurance type, and total cost. The linkage of registries with claim databases offers a valuable opportunity to explore associations between cancer patient characteristics, demographic groups, evolving FP treatment costs and insurance coverage, and the utilization of FP services.

## 8. Conclusions

Insurance mandates for medical FP are new and differ substantially in their coverage depending on the state. Given that the first state mandate was recently passed in 2017, there are limited data on how the utilization of FP services will be impacted. Preliminary data demonstrate increased use of FP services. It is crucial to continue investigating the impacts of these mandates on equitable care for cancer patients as well as patients with other medical indications for FP and to convince additional states to advocate for these mandates. Other areas of research to improve the reach of these mandates include developing interventions that ensure the provision of equitable and timely FP counseling and referrals as well as social service support for patients navigating FP services, such as insurance benefits. Insurance policies are complex, and without financial counseling, a patient with insurance benefits may not understand how to access FP services.

## 9. Future Directions

The scarcity of data constitutes the largest barrier in our current understanding of the effects of insurance mandates on the utilization of FP services. Further research is necessary to comprehend the evolving trends and long-term impact of insurance mandates and other forms of coverage or assistance on FP referrals, FP utilization, and demographic groups, within the United States as well as across other countries. It is imperative to continue collecting state-specific data on individuals that access FP services both pre- and post-mandates. Given the limited number of patients accessing FP services, there is a need to aggregate data across multiple states and examine outcomes on a larger scale. Collaborative, multi-site research endeavors are encouraged and will necessitate access to cancer registry databases. The linkage of state-wide all-payer claim databases with cancer registries will allow for an analysis of cancer patient characteristics that may impact the utilization of FP services. Advocacy efforts will be pivotal in swiftly implementing insurance mandates across all U.S. states, persuading U.S. states to cover noncommercial insurance plans, and assisting patients in navigating the financial aspects of insurance coverage, ultimately improving FP access across demographic groups.

## Figures and Tables

**Table 1:** Fertility Preservation Insurance Mandates by State.

U.S. State	Year that the Mandate Was Signed into Law	Insurance Coverage	FP Coverage
California	2019	Commercial	Iatrogenic cases of FP
Colorado	2020	Commercial	Iatrogenic cases of FP
Connecticut	2017	Commercial	Iatrogenic cases of FP
Delaware	2018	Commercial	Iatrogenic cases of FP
Illinois	2018	Commercial and noncommercial	Iatrogenic cases of FP
Kentucky	2023	Commercial, state-employee health plan, and state post-secondary education institutions	Iatrogenic cases of FP
Louisiana	2023	Commercial	Iatrogenic cases of FP
Maine	2022	Commercial	Iatrogenic cases of FP
Maryland	2018	Commercial	Iatrogenic cases of FP
Montana	2023	Commercial and noncommercial	Iatrogenic cases of FP with an active cancer diagnosis
New Hampshire	2019	Commercial	Iatrogenic cases of FP
New Jersey	2020	Commercial, state health benefits, school employee health benefits	Iatrogenic cases of FP
New York	2019	Commercial	Iatrogenic cases of FP
Rhode Island	2017	Commercial	Iatrogenic cases of FP
Texas	2023	Commercial	Iatrogenic cases of FP with an active cancer diagnosis
Utah	2021	Commercial and noncommercial	Iatrogenic cases of FP with an active cancer diagnosis

## Data Availability

All data is available in referenced articles and referenced legislation.

## Appendix A

Search algorithm PubMed: ((“Fertility Preservation”[MeSH Terms] OR “Fertility Preservation”[All Fields] OR (“fertil*”[Text Word] AND “preserv*”[Text Word])) AND (“Patient Acceptance of Health Care”[MeSH Terms] OR “Health Services Accessibility”[MeSH Terms] OR “Insurance”[MeSH Terms] OR “Insurance Coverage”[MeSH Terms] OR “insurance, health”[MeSH Terms] OR “Insurance”[Text Word] OR “insur*”[Text Word] OR “cover*”[Text Word] OR “policy”[Text Word] OR “premium*”[Text Word] OR “health plan”[Text Word] OR “utili*”[Text Word] OR “access*”[Text Word] OR (“Legislation as Topic”[MeSH Terms] OR “law”[Text Word] OR “legislat*”[Text Word] OR “mandat*”[Text Word] OR “statute*”[Text Word] OR “bill”[Text Word] OR “require*”[Text Word]))) AND ((english[Filter]) AND (2017:2022[pdat])).
